# Fabrication of Graphene-Modified Styrene–Acrylic Emulsion by In Situ Aqueous Polymerization

**DOI:** 10.3390/polym14183763

**Published:** 2022-09-08

**Authors:** Yalin Li, Jieling Luo, Baoquan Huang, Hongjun Jin, Xiaoli Sun, Changlin Cao, Qinghua Chen, Qingrong Qian

**Affiliations:** 1 College of Environmental and Resource Sciences, Fujian Normal University, Fuzhou 350007, China; 2 Engineering Research Center of Polymer Green Recycling of Ministry of Education, Fuzhou 350007, China; 3 Fujian Key Laboratory of Pollution Control & Resource Reuse, Fuzhou 350007, China

**Keywords:** graphene, aqueous dispersions, in situ polymerization, modified styrene–acrylic emulsion

## Abstract

With the aim of developing green coatings, styrene–acrylic emulsion has been widely used in architectural coatings due to its excellent environmental protection and energy conservation. Nevertheless, the lack of water and oxygen resistance of water-based styrofoam coatings has promoted various nanomaterials being studied for modification. To improve the performance of waterborne styrofoam coating, we introduced the graphene nanopowder and expected to enable it with the function of electromagnetic interference (EMI) shielding to reduce the damage of electromagnetic radiation. In this paper, the problem of poor interface compatibility between graphene and polymer resin was successfully addressed by in situ polymerization. In the process of pre-polymerization of styrene–acrylic emulsion monomer, graphene-modified styrene–acrylic emulsion was obtained by introducing graphene aqueous dispersion. The results showed that the styrene–acrylic emulsion with 4 wt% aqueous graphene dispersions exhibited the best dispersion stability, improved water and oxygen resistance, and the conductivity reached 1.89 × 10^−2^ S/cm. Then, the graphene-modified coating for building was prepared by using graphene-modified styrofoam emulsion. All the performance indexes of the coating are in line with the industry standards, and it still showed benign EMI shielding effect even when the graphene content was low. It is demonstrated that in situ polymerization technology and the application of graphene in resin coatings modification will promote the development of green coatings.

## 1. Introduction

Owing to the global energy crisis and environmental pollution, one of the overarching goals for the worldwide scientific community is to develop an environmentally friendly society. In the field of green coatings, waterborne coatings which contain no or only a small amount of organic solvents, have the great advantages of low pollution, being tasteless, and causing little or almost no harm to human health and the environment [1,2,3]. Thus, the development of eco-friendly waterborne coatings has become a major trend in coatings [4,5,6,7]. Styrene–acrylic emulsion, obtained by copolymerization of acrylate and styrene monomer in aqueous solution, and showing superior adhesion, alkali resistance, scrub resistance and environmental friendliness, has aroused great attention in the fields of coatings [8,9,10], inks [11] and adhesives [8,9,10,12]. However, there still remain challenges to replace the high-performance solvent-borne coatings due to the hydrophilicity of waterborne resin and the micropores between the uncrosslinked resin domains, which result in poor water and oxygen resistance, and greatly weaken the performance of styrene–acrylic coatings [8,13]. To circumvent these defects, many nanomaterials such as TiO_2_, SiO_2_ and ZnO have been incorporated to modify the waterborne coatings and improve the thermal, rheological, mechanical and anticorrosion performance [14,15,16]. Moreover, the ubiquitous electromagnetic radiation is endangering human health, as well as the performance and life of electronic components [17,18], so electromagnetic interference (EMI) shielding in the application of cold coatings has received certain attention [19].

Compared with traditional metal-based EMI shielding materials, the excellent properties of conductive polymer composites include electrical conductivity, chemical and temperature resistance, light weight, high specific surface area and large aspect ratio, which are all conducive to the application of EMI shielding [19]. Among them, graphene with its unique structure and properties has been widely used as nanofiller in polymer composites since the report by Geim and co-workers [20], and has dramatically achieved material performance improvements, including EMI shielding [21]. For example, Yu and co-workers prepared functional polymethylmethacrylate (PMMA)-graphene nanocomposites with excellent electrical conductivity and improved EMI shielding performance by subcritical CO_2_ foaming technique [22]. Bontas et al. [23] modified graphene with HMDA successfully compatible with the epoxy resin components. The epoxy nanocomposites based on Hexamethylene Diamine-modified graphene displayed better flexibility along with an identical EMI shielding effect when compared to the unmodified graphene. Their analysis showed that the pristine graphene sheets are highly unstable in the polymer matrix owing to its high surface energy [24]. The graphene sheets are prone to agglomerating and precipitating through the strong π-π stacking between layers, resulting in poor interfacial compatibility with polymer resin, which poses a major obstacle for the modification of graphene with polymer [25,26]. Therefore, synthesis methods for combining graphene with polymers have also been studied in depth. For example, Li and co-workers [27] synthesized a sulfonic acid groups grafted graphene oxide, sulfonated graphene (SG), which can be stably dispersed in water and aqueous epoxy emulsion at a relatively high filler content. When the SG content reaches 1.0 wt%, the most significant improvement in impedance modulus and corrosion protection properties was achieved due to the high stability of the emulsion and the physical barrier properties of the abundant SG. Since the improvement of graphene dispersion relies on enhanced ultrasound and complex chemical modification [27,28], it is of great significance to design a simple technique for composite preparation of graphene and polymers.

This work uniquely introduced in situ polymerization which provides an ideal strategy to overcome the interfacial incompatibility between graphene and the polymer resins; surfactants were added to improve the dispersion of graphene in water; and it successfully realized an eco-friendly method of preparing graphene-modified styrene–acrylic emulsion in aqueous solution. The results revealed that graphene modification can effectively improve the rheological properties of the styrene–acrylic emulsion along with the excellent EMI shielding effect. Finally, this graphene-modified styrene–acrylic emulsion was successfully applied as a waterborne architectural coating which shows excellent water resistance and low water permeability. The successful modification of graphene also affords the coating with good electromagnetic shielding performance. Each performance index of the graphene-modified styrene–acrylic emulsion-based coating reaches the industry standard (T/CNCIA 01004-2017) for application as waterborne graphene electromagnetic shielding coating for architecture. We expect the strategy will open up a new vista in fabricating graphene-modified styrene–acrylic emulsion and the application of waterborne coatings.

## 2. Materials and Methods

### 2.1. Materials

Graphene (mean diameter: 4–7 μm, thickness: 1–3 layers, purity: 93%, diameter-thickness ratio: 8500, bulk density: 0.01–0.02 g/mL, KNG-G2-3), sodium carboxymethyl cellulose (CMC, η = 6000 mPa·s), Polyvinyl alcohol and sodium ligninsulfonate (SLS) were purchased from Xiamen Kaina Co., Clariant Chemicals Co., Ltd. (Guangzhou, China) and Aladdin Reagents Co., respectively. Styrene (St), butyl acrylate (BA), methyl methacrylate (MMA), propyl methacrylate (n-PMA), alkoxyphenol ethoxylate (emulsifier, OP-10), sodium polymethacrylate (protective gel, PMA), ammonium persulfate (APS) and sodium bicarbonate (NaHCO_3_) were all purchased from Chemical Reagent Co., Ltd. (Shanghai, China) with A.R. grade. Ethylene glycol, Thickening agent HS300, Dispersing agent P-19, Wetting agent X-405, Titanium dioxide, White clay (4000 mesh), CaCO_3_ (1250 mesh), Hydroxyethyl cellulose (HEC), Coalescing agents, Flatting agent 3020, Antifoaming agents 3016, Thickening agent ASE-60 and Corrosion remover 981 were all provided from Fuzhou Aidite New Material Technology Co., Ltd. (Fujian, China). All the above chemicals were used as received without further purification.

### 2.2. Experimental Method

#### 2.2.1. Preparation of Aqueous Graphene Dispersion

The stable aqueous graphene dispersion with high content was made with the aid of polyvinyl alcohol (PVA) and sodium carboxymethylcellulose (CMC) as stabilizer through the following process [29]. First, the stabilizer solution was prepared by adding a certain amount (the weight ratio of stabilizer and graphene is in the range of 50% to 80%) of PVA and CMC to 300 mL of deionized water and stirring uniformly. Second, 15 g of commercial graphene was added to two-thirds of the stabilizer solution, manually stirred until the graphene was completely wetted. Then, the mixture was transferred to a sand mill and ground for 2 h at a speed of 2000 r/min. Finally, the remaining one-third of the stabilizer solution was added to the sand mill and ground for another 1 h to obtain a high concentration aqueous graphene dispersion. Exploration experiments show that, in the graphene dispersion solution with the same amount of SLS, PVA, CMC, PVA/CMC, PVA/SLS or CMC/SLS, the Zeta potential value of graphene aqueous dispersions under the action of PVA/CMC was the highest. The content of PVA and CMC was optimized to get a stable dispersion, which was 2.67 wt% and 1.33 wt%, respectively. The content of graphene in the prepared aqueous solution is up to 4.57% and used in the following experiments.

#### 2.2.2. Monomer Pre-Emulsification

Distilled water (150 g) and OP-10 (26 g) were added to a three-necked flask, and then stirred at high speed until foaming. Four kinds of monomers, St (200 g), MMA (50 g), BA (210 g) and n-PMA (9 g), were mixed and added to the foamed emulsifier solution within 30 min. Then the aqueous graphene dispersion was continuously added at the same speed. The obtained mixture was stirred at high speed for 20 min, and then stirred at low speed for 10 min to obtain a pre-emulsion.

#### 2.2.3. Semi-Continuous Emulsion Polymerization

Deionized water (220 g), OP-10 (4 g) and PMA (15 g) were added to a four-necked flask in sequence and stirred at high speed until foaming. One-third of the pre-emulsion and the initiator–buffer mixed solution (1.6 g APS and 0.2 g NaHCO_3_ in 12 g deionized water) were added to above mixture at 70 °C. Then the remaining pre-emulsion and the initiator–buffer mixed solution (1.5 g APS and 1.5 g NaHCO_3_ in 80 g deionized water) were dripped in within 3 h. Finally, an extra dose of initiator solution (0.6 g APS in 10 g deionized water) was continuously added dropwise at the same rate to produce a sufficient polymerization. Cool down naturally after 2 h of reaction.

#### 2.2.4. Preparation of Aqueous Graphene-Modified Styrene–Acrylic Emulsion

The pH of the above emulsion was adjusted to 7.5~8.0 with ammonia water when the temperature cooled to 40 °C. The product was filtered with a 100-mesh filter cloth to obtain an aqueous graphene-modified styrene–acrylic emulsion.

#### 2.2.5. Preparation of Graphene-Modified Styrene–Acrylic Architectural Coating

The aqueous graphene-modified styrene–acrylic emulsion-based coating was prepared according to the following formulation and an industrial process (Table 1).

(1) The preparation of graphene-modified styrene–acrylic emulsion architectural coatings requires the preparation of premixed slurry; according to the ratio of premixed slurry shown on the left of Table 1, the drugs and auxiliaries are weighed. CaCO_3_, White clay, Titanium dioxide, HEC and wetting agent X-405 are added to the dispersion cylinder, and joined two-thirds of distilled water for premixed, and the mixture was stirred at a speed of 500 r/min for 10 min to moisten the filler and water fully. Ethylene glycol, dispersant and thickener were added to the dispersion cylinder in turn, and the mixture was stirred at a speed of 1500 r/min for 30 min. The remaining deionized water was added and the mixture was stirred for 20 min to obtain premixed slurry.

(2) According to the ratio on the right of Table 1, the graphene-modified styrene–acrylic emulsion was added to the premix slurry, followed by coalescing agents, flatting agent, corrosion remover, thickening agent, antifoaming agents and deionized water. The mixture was stirred at a speed of 2000 r/min for 40 min to obtain graphene-modified styrene–acrylic emulsion composite coating.

Among the above, thickening agent HS300 and thickening agent ASE-60 are increasing the viscosity of the coating, so it can better adhere to the substrate material. Dispersing agent P-19 is used to disperse the particles which need to be dispersed in waterborne coatings on the surface, so that it has better stability. Wetting agent X-405 is to moisten the substrate. Titanium dioxide, White clay (4000 mesh) and CaCO3 (1250 mesh) are fillers. Hydroxyethyl cellulose (HEC) can act as a binder and thickener. Coalescing agents are to make the coating better film. The purpose of adding flatting agent 3020 is to make the fluidity of the coating better and smoother. In the process of high-speed stirring, samples will be bubbles, so it needs to be anti-foamed by agent 3016 to stir more fully and reduce the production of bubbles after coating film.

The graphene-modified styrene–acrylic architectural coating is denoted as G-St/Ac-C. The coating without graphene (St/Ac-C) was also prepared for comparison.

### 2.3. Characterization

The X-ray Diffraction (XRD) patterns were performed on a Bruker D8 diffractometer with Cu-Kα radiation (λ = 0.15406 nm) over a range of 10–80°. The field-emission scanning electron microscope (SEM, Hitachi 8100) and transmission electron microscopy (TEM, FEI F20 S-TWIN) were carried out to analyze the morphologies and microstructures of the graphene in the styrene–acrylic emulsion. Confocal Raman microspectrometer (DXR2xi) with an Arion laser at the excitation wavelength of 532 nm was used to collect Raman spectra of the samples. To confirm the amount of carbon in the nanocomposites, thermogravimetric analysis (TGA) was performed (TGA Q50, TA, New Castel, DE, USA) in air atmosphere. Fourier-transform infrared (FTIR) spectra were obtained using a Thermo Fisher Scientific Instruments Nicolet iS10 FTIR spectrometer at room temperature in the wavenumber range of 400–4000 cm^−1^. Rheological properties of styrene–acrylic emulsion were measured using a rheometer (DHR-2, Waters, Wakefield, MA, USA). A 40 mm stainless steel parallel plate was used for the tests and the plate gap was 1 mm. All experiments were carried out at a constant temperature of 25 °C. The Zeta potential of aqueous graphene dispersion was measured using a Malvern Zetaszier Nano-ZS; the instrument measures the particle size by the principle of Dynamic Light Scattering (DLS). Before the test, the sample was diluted with deionized water to a certain multiple before measurement. Surface resistivity was measured by using a four-probe setup (RTS-9, Guangzhou Sitanzhen Technology Co., Guangzhou, China). The electromagnetic shielding efficiency for graphene-modified styrene–acrylic architectural coating was measured with a shielding effectiveness tester (DR-S02) using the flange coaxial test device for shielding effectiveness.

## 3. Results and Discussion

**Scheme 1:**
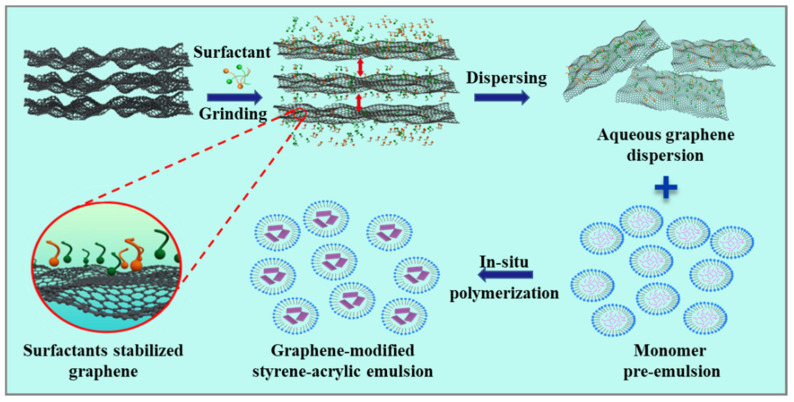
The schematic illustration of in situ graphene modification and styrene–acrylic polymerization.

Scheme 1 shows the schematic process of the facile strategy for fabricating graphene-modified styrene–acrylic emulsion through in situ modification and polymerization in aqueous solution, which mainly contains three steps of graphene dispersion, monomer pre-emulsion and in situ polymerization. Owing to the strong π-π interactions between layers, the hydrophobic graphene is unstable when dispersed in water. In order to improve the stability of aqueous graphene dispersion, sustainable polymeric surfactants PVA, CMC and SLS were utilized. Surfactants are prone to binding on the surfaces of graphene to turn the high energy surfaces into relatively low ones, which can greatly improve the thermodynamic stability. Meanwhile, the ionic surfactants endow the graphene with net surface charges, which also can effectively inhibit the agglomeration and precipitation of graphene and remarkably improve the kinetic stability.

**Figure 1:**
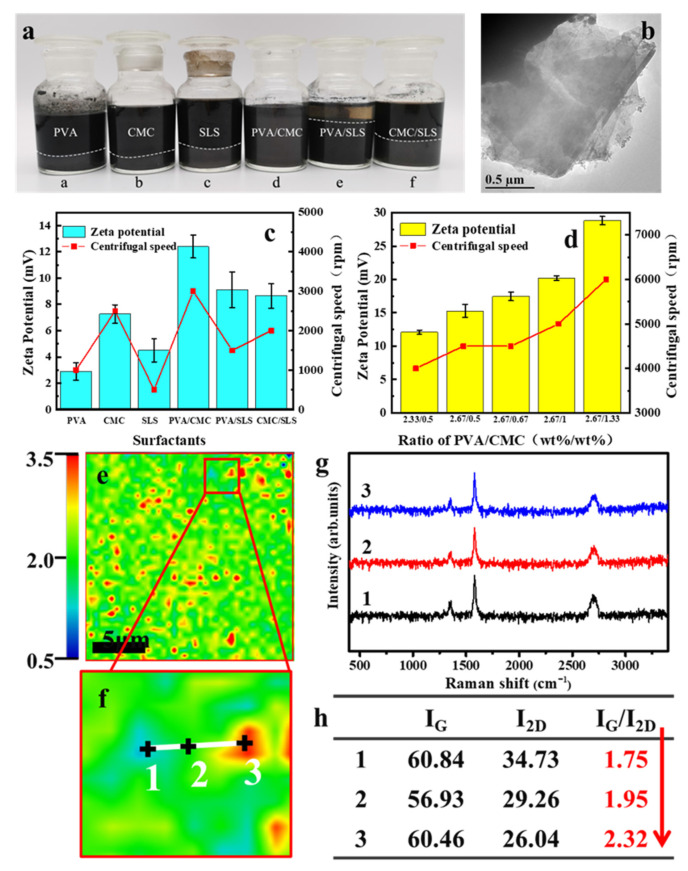
(**a**) Photos of the 5 wt% graphene dispersions with different surfactant including PVA (3 wt%), CMC (3 wt%), SLS (3 wt%), PVA/CMC (1.5 wt%:1.5 wt%), PVA/SLS (1.5 wt%:1.5 wt%) and CMC/SLS (1.5 wt%:1.5 wt%), respectively. The pictures were taken 2 months after sample preparation. (**b**) The TEM image of the graphene dispersion with PVA/CMC (1.5 wt%:1.5 wt%). (**c**) Zeta potential (cyan histogram) and the highest centrifugation speed before precipitation (red line) for the fresh samples in (**a**). (**d**) Zeta potential (yellow histogram) and the highest centrifugation speed before precipitation (red line) for the fresh samples with 5 wt% graphene and different proportions of PVA/CMC which are 2.33 wt%/0.5 wt%, 2.67 wt%/0.5 wt%, 2.67 wt%/0.67 wt%, 2.67 wt%/1 wt%, and 2.67 wt%/1.33 wt%, respectively. (**e**) Raman mapping of the graphene dispersion with PVA/CMC (2.67 wt%:1.33 wt%). (**f**) The enlarged view of the selected part of (**e**). (**g**) The Raman spectrums of the three representative sites in (**f**). (**h**) Intensity of the corresponding G-band and 2D-band in (**g**) and the I_G_/I_2D_ values.

Thus, with the aid of 3 wt% surfactants including PVA (3 wt%), CMC (3 wt%), SLS (3 wt%), PVA/CMC (1.5 wt%:1.5 wt%), PVA/SLS (1.5 wt%:1.5 wt%) and CMC/SLS (1.5 wt%:1.5 wt%), the stability of the dispersion with 5 wt% graphene is remarkably improved. In contrast, when 5 wt% graphene was dispersed in pure water, it almost completely stratifies within one week. Noticeably different from the other surfactant systems with distinct precipitation after two months, the dispersion with PVA/CMC shows little stratification, indicating excellent stability (Figure 1a). Transmission electron microscopy (TEM) shows the graphene display disordered arrangement with no obvious agglomeration in the dispersion (Figure 1b). Meanwhile, after centrifugated at 3000 rpm for 5 min, the graphene/PVA/CMC dispersion is still homodispersed. However, the other systems are all stratified. The highest centrifugation speed before precipitation for each system is shown in Figure 1c (red line). In line with this result, with the addition of PVA/CMC, the graphene dispersion shows the highest absolute value of Zeta potential (Figure 1d, −12.31 mV), which is beneficial to the kinetic stability of the graphene dispersion. Thus, in order to optimize the kinetic stability, the ratio of PVA/CMC was systematically adjusted. As shown in Figure 1d (yellow histogram), the absolute value of Zeta potential increased in line with the increasing of CMC content, due to the increasing net surface charges. Interestingly, when the content of CMC is fixed at 0.5 wt%, the absolute value of the Zeta potential also increased with the increasing of PVA content. This may be ascribed to the H-bonding between PVA and CMC, which can facilitate the binding of charged CMC to graphene surfaces. When the ratio of PVA/CMC reaches 2.67 wt%/1.33 wt%, the absolute value of Zeta potential increases to 28.83 mV, which is about 30 mV. Thus, the dispersion was supposed to show optimal kinetic stability. As Figure 1d (red line) shows, the highest centrifugation speed before precipitation also increases with the absolute value of Zeta potential. In the PVA/CMC (2.67 wt%/1.33 wt%) dispersion, the graphene is homodispersed even after centrifugation at 6000 rpm for 5 min, due to the remarkable stability. In order to figure out the dispersion state of graphene layers in the dispersion, Raman mapping was performed. Figure 1e shows the Raman surface scan diagram of the dispersion with PVA/CMC (2.67 wt%/1.33 wt%) and Figure 1f is the enlarged view of the selected part with three distinct sites. The corresponding Raman spectrums are shown in Figure 1g for the three representative sites. According to the literature, the peaks located at 1580 and 2700 cm^−1^ are corresponding to the characteristic peaks of G-band and 2D-band of graphene, respectively [30,31,32]. The relative intensity of I_G_/I_2D_ value reveals the number of graphene layers. Generally, graphene with an I_G_/I_2D_ about 0.5~1 contains 1–2 layers, while multiple layers when I_G_/I_2D_ = 1.5~3. Figure 1d shows the I_G_/I_2D_ values of the three sites which indicates the graphene stacks with multiple layers other than bulk graphite. Thus, in the following polymerization process, we chose the well-stabilized aqueous graphene dispersion with PVA/CMC (2.67 wt%:1.33 wt%) to modify the styrene–acrylic emulsion.

**Figure 2:**
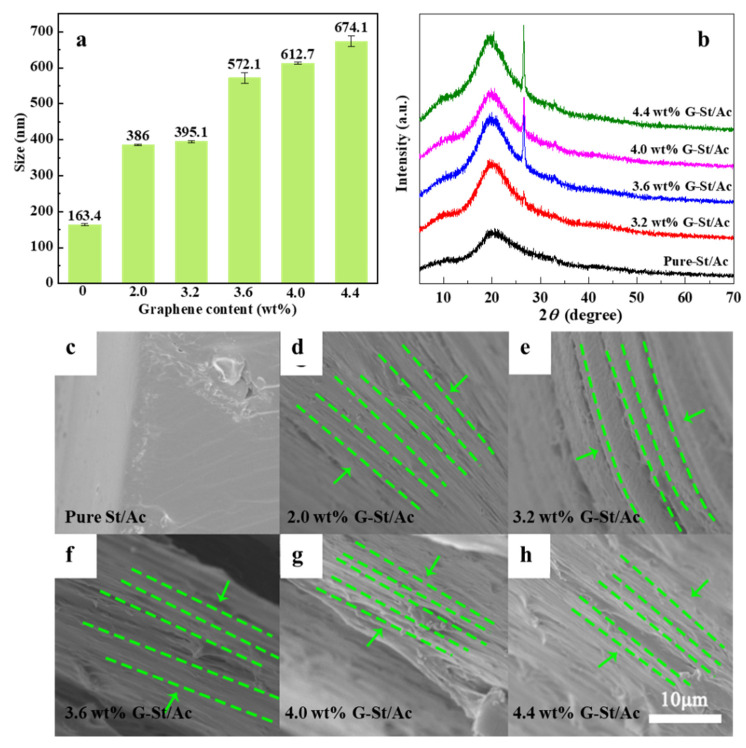
The particle size (**a**), XRD patterns (**b**) and SEM images (**c–h**) of the graphene-modified styrene–acrylic emulsion with different amounts of graphene dispersion addition.

Dynamic light scattering (DLS), X-ray diffraction (XRD) and transmission electron microscopy (TEM) were performed to identify the successful modification of graphene. Compared to the pure styrene–acrylic emulsion, DLS results show that with the addition of graphene, the size of the particles in the emulsion increases in line with the increasing of graphene addition (Figure 2a). Furthermore, the graphene-modified styrene–acrylic emulsion was cast onto a polytetrafluoroethylene (PTFE) mold and a composite thin film with thickness of 0.5–1 mm was obtained after the evaporation of water at 55 °C for 8 h. The XRD patterns (Figure 2b) show that with the increase of graphene addition, the intensity of the characteristic peak of graphene at 2*θ* = 26° increases. Compared with the film formed from the pure styrene–acrylic emulsion (Figure 2c), the films with graphene addition (Figure 2d–h) show clear layered graphene structure. The styrene–acrylic polymer is supposed to be polymerized on the surface of the graphene and inserted into the graphene sheets to stabilize the emulsion through preventing π-π stacking. Furthermore, this unique layered structure provides physical barriers to prevent the penetration of oxygen and water, which may endow the emulsion with excellent corrosion resistance [8].

**Figure 3:**
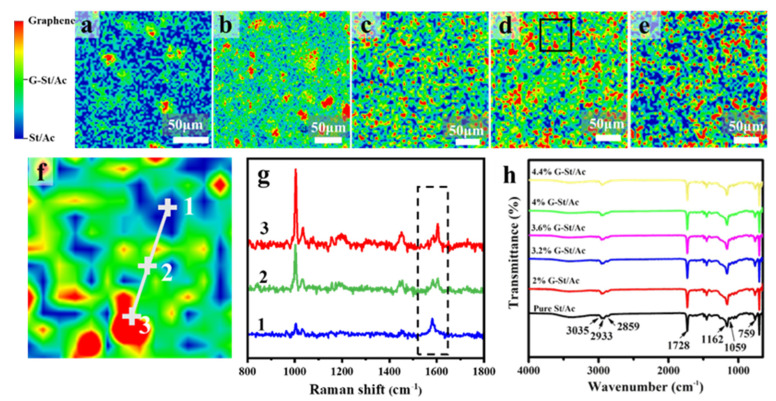
(**a**–**e**) Raman mapping of the graphene-modified styrene–acrylic emulsion with 2.0, 3.2, 3.6, 4.0 and 4.4 wt% addition of the aqueous graphene dispersion. (**f**) The enlarged view of the selected part of (**d**). (**g**) The Raman spectrums of the three representative sites in (**f**). (**h**) The FT-IR spectrums of the graphene-modified styrene–acrylic emulsions with different amounts of graphene dispersion addition.

Raman mapping was performed to characterize the dispersion state and the relative content of graphene in the emulsion. Figure 3a–e are the Raman surface scan diagrams of the emulsions with different graphene additions of 2.0 wt%, 3.2 wt%, 3.6 wt%, 4.0 wt% and 4.4 wt%, respectively. Figure 3f is the enlarged view of the selected part of Figure 3d and the Raman spectrums are shown in Figure 3g for three representative sites. According to the literature, the peak at 1600 cm^−1^ is assigned to styrene–acrylic emulsions [33]. Thus, the ratio of I_1580_/I_1600_ reveals the graphene content at the corresponding site. As Figure 3g shows, the ratio of I_1580_/I_1600_ gradually decrease from site 3 to site 1, and there is almost no graphene dispersion at site 1 in the blue area. In Figure 3a, the graphene addition is relatively low (2.0 wt%), so a large blue area exists. As the addition increases to 3.2 wt%, 3.6 wt% and 4.0 wt%, the graphene dispersed uniformly in the emulsion (Figure 3b–d). However, when the addition reaches 4.4 wt%, there are many pure styrene–acrylic regions separated (Figure 3e), which can be ascribed to the excessive addition of graphene resulting in agglomeration and phase separation. Figure 3h shows the attenuated total internal reflectance Fourier transform infrared spectroscopy (ATR-FTIR) results of the emulsions with different graphene additions. They display a series of peaks at 2859–3035, 1728, 1243 and 1162 cm^−1^ which are assigned to the vibration of -CH_3_, -CH_2_, C=O, C-O, respectively, and the peaks at 759–1059 cm^−1^ are characteristic peaks of butyl acrylate, which are consistent with the literature results [33]. Thus, the in situ polymerization of the styrene–acrylic emulsions has been successfully achieved in aqueous solution with graphene modification.

**Figure 4:**
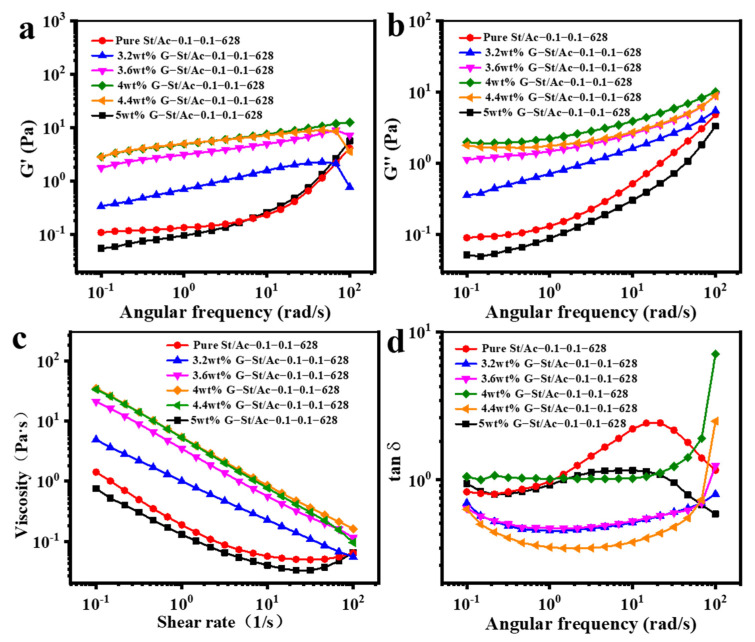
(**a**) Dependence of storage modulus (G′) on strain. (**b**) dependence of loss modulus (G′′) on strain, (**c**) complex viscosity versus angular frequency, and (**d**) dependence of loss tangent (tanδ) on strain, for the styrene–acrylic emulsion with different addition amounts of aqueous graphene dispersion.

For coatings, the rheological properties are critical. Thus, the rheological properties of the obtained emulsions with different graphene content were investigated (Figure 4a–d). Figure 4a,b show the relationship between the storage modulus (G′) and loss modulus (G″) with frequency (ω) of the different added amounts of aqueous graphene. When adding 3.6~4.4 wt% aqueous graphene dispersion into the styrene–acrylic emulsion, there is a wider plateau modulus at low frequencies, and the G′ value of the addition of 4 wt% aqueous graphene dispersion is higher than other samples (Figure 4a). Figure 4d shows the relationship between viscosity (v) and frequency (ω) for different added amounts of aqueous graphene. The viscosity of the sample appears as shear thinning behavior in the low frequency region, and shear thickening occurs in the high frequency region, which can be ascribed to the structure between the graphene sheet and the emulsion molecule in the high frequency region being destroyed. Figure 4d displays the curves of tanδ versus frequency (ω) for the different added amounts of aqueous graphene. It is clear that the styrene–acrylic emulsion with graphene aqueous dispersion was gentlest with the addition of 3.2 wt%. When adding 3.6~4.4 wt% of aqueous graphene into styrene–acrylic emulsion, the modified styrene–acrylic emulsion was stable. In the low frequency region, tanδ < 1 shows that the elasticity of the material is greater than the viscosity. Therefore, the modified styrene–acrylic emulsion possesses the optimal stability of dispersion when the aqueous graphene is added at 4 wt%.

**Figure 5:**
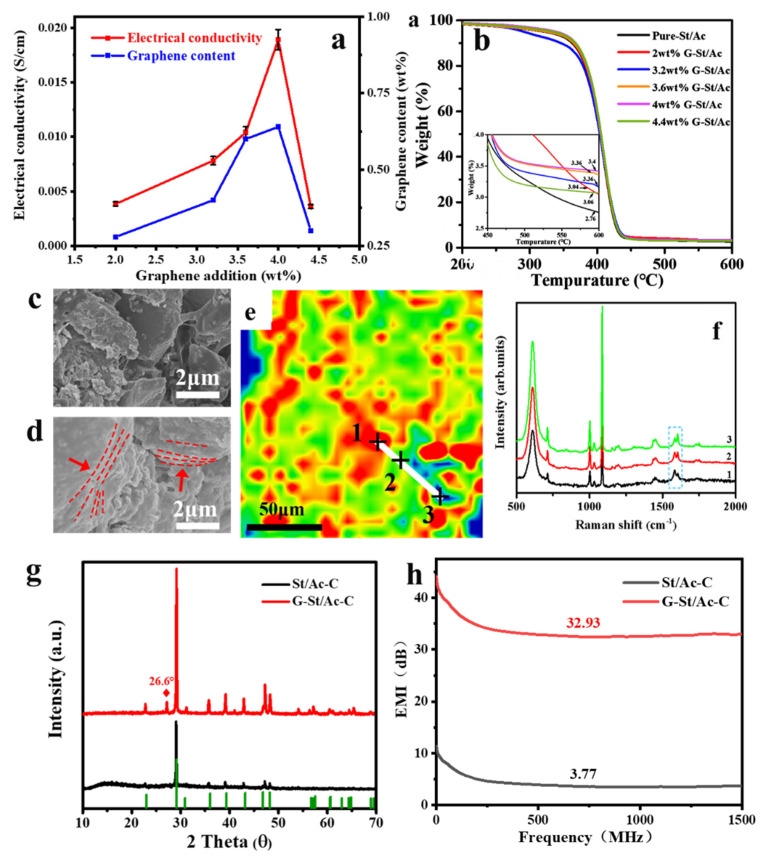
(**a**) The electrical conductivity (red line) and the exact graphene content (blue line), (**b**) the TGA curves of the graphene-modified styrene–acrylic emulsions with different amounts of graphene dispersion addition, (**c**) SEM images of St/Ac-C, (**d**) SEM images of G-St/Ac-C, (**e**) Raman mapping of G-St/Ac-C, (**f**) Raman spectrum of G-St/Ac-C, (**g**) XRD patterns of St/Ac-C and G-St/Ac-C, and (**h**) EMI shielding efficiency of St/Ac-C and G-St/Ac-C.

Compared to the traditional nonconductive styrene–acrylic emulsion, the loading of graphene endows the modified emulsion with good electrical conductivity. Figure 5a shows that the conductivity increases in line with the increasing graphene addition and gets the highest value, 1.89 × 10^−2^ S/cm, when the addition reaches 4.0 wt%. Then, the conductivity sharply drops to 3.62 × 10^−3^ S/cm at the addition of 4.4 wt%. In order to figure out the mechanism behind the conductivity change with the graphene addition, the exact graphene content in the graphene-modified styrene–acrylic emulsion was investigated by thermogravimetric analysis (TGA), SEM topography and Raman mapping image. As shown in Figure 5b, the residual weight of the pure styrene–acrylic emulsion is 2.76%. The residual weights of the emulsions with 2.0 to 4.4 wt% of graphene addition are 3.04%, 3.16%, 3.36%, 3.40% and 3.06%, respectively. Therefore, the exact graphene contents were calculated to 0.28%, 0.40%, 0.60%, 0.64%, and 0.30%, respectively. It shows that the graphene content is positively correlated with the conductivity (Figure 5a). When 4.0 wt% graphene dispersion is added, the exact graphene content in the modified styrene–acrylic emulsion reaches the peak, which is well consistent with the highest conductivity. And both the exact graphene content and the conductivity of 4.4 wt% addition is close to the 2.0 wt% system, indicating that conductivity depends on the exact graphene content in the emulsion. Furthermore, although distinct phase separation occurs in the 4.4 wt% system with many discrete styrene–acrylic regions (Figure 3e), the conductive graphene regions are still continuous, which maintains the conductivity at the same level with the 2.0 wt% system.

To verify the practical application as a waterborne coating, the graphene-modified styrene–acrylic architectural coating (G-St/Ac-C) was prepared according to the industrial formulation (Table 1). Amazingly, the G-St/Ac-C displays excellent water resistance and low water permeability, which may be ascribed to the physical barrier properties provided by graphene layers. Comparing it with the St/Ac-C, the surface of the G-St/Ac-C is smoother with obvious layered structure inside (Figure 5c,d). In Raman mapping (Figure 5e), the same blue at 3 is less frequent, while red and green are more frequent, so it can be seen that graphene is uniformly distributed. And most of the red and green areas can be connected into a complete area, indicating that graphene has formed a complete conductive path inside the paint, which is also conducive to electromagnetic shielding properties. The corresponding Raman spectrum (Figure 5f) indicates that the amount of graphene decreases. Meanwhile, the XRD patterns displayed in Figure 5g show that the diffraction intensity of CaCO_3_ is distinctly improved in the G-St/Ac-C, indicating that the addition of graphene may facilitate the crystallization of CaCO_3_ to form more ordered arrangement. CaCO_3_, as a main filler, has an important influence on the properties of the architectural coating. The enhanced crystallinity of CaCO_3_ may also be beneficial to improve water resistance and reduce water permeability. Furthermore, Figure 5h shows the electromagnetic shielding efficiency curves of St/AC-C and G-ST/AC-C. The electromagnetic shielding efficiency value of St/AC-C samples without graphene is only 3.77 dB in the high-frequency region, and there is almost no shielding efficiency. On the contrary, the G-ST/AC-C electromagnetic shielding efficiency with graphene is greatly increased, reaching 32.93 dB at high frequencies. The introduction of graphene endows the coating with special electromagnetic shielding performance (Figure 5h). According to the industry standard (T/CNCIA 01004-2017) for waterborne graphene electromagnetic shielding coating for architecture, each performance index of the G-St/Ac-C meets the requirement. Thus, the presented aqueous G-St/Ac-C is very promising for practical application.

## 4. Conclusions

In this study, aqueous graphene dispersion with high stability was readily prepared and the graphene-modified styrene–acrylic emulsion was successfully synthesized by in situ polymerization in aqueous solution through a facile eco-friendly method. The graphene was first dispersed into aqueous solution with the aid of polymeric surfactant stabilizer PVA and CMC. Then the aqueous graphene dispersion was added to the pre-emulsion with monomers and initiator. Through in situ polymerization, the graphene was successfully loaded into the styrene–acrylic emulsion. The optimum graphene addition amount (4.0 wt%) was explored through systematically investigating the rheological properties, conductive properties and the exact graphene content with different graphene additions. The graphene modification improved the rheological properties of the styrene–acrylic emulsion and endowed it with excellent electrical conductivity. The emulsion was further applied as graphene electromagnetic shielding coating for architecture and every performance reaches the industry standard. We hope this in situ polymerization method for graphene-modified styrene–acrylic emulsions will be a potential application in the field of environmentally friendly coatings.

## Figures and Tables

**Table 1:** Formulation of the graphene-modified styrene–acrylic architectural coating.

Raw Materials	wt%	Raw Materials	wt%
Water	22	Water	2.4
Ethylene glycol	1	(Graphene-modified) styrene–acrylic emulsion	34
Thickening agent HS300	0.3	Coalescing agents	1
Dispersing agent P-19	0.7	Flatting agent 3020	0.15
Wetting agent X-405	0.3	Antifoaming agents 3016	0.15
Titanium dioxide	2.5	Thickening agent ASE-60	0.2
White clay (4000 mesh)	4	Corrosion remover 981	0.1
CaCO_3_ (1250 mesh)	32	Total: 100.9	
Hydroxyethyl cellulose (HEC)	0.17		
Pre-mixed slurry	79.97		

## Data Availability

Not applicable.
